# Induction of Angiogenesis and Neovascularization in Adjacent Tissue of Plasma-Collagen–Coated Silicone Implants

**Published:** 2010-09-28

**Authors:** Andrej Ring, Stefan Langer, Daniel Tilkorn, Ole Goertz, Lena Henrich, Ingo Stricker, Hans-Ulrich Steinau, Lars Steinstraesser, Joerg Hauser

**Affiliations:** ^a^Department of Plastic and Hand Surgery, University Hospital Bergmannsheil Bochum, Germany; ^b^Institute of Pathology, Ruhr University Bochum, Germany

## Abstract

**Objective:** Formation of encapsulating, avascular fibrous tissue is deemed to decrease implant's biocompatibility and versatility. We investigated whether plasma-mediated collagen coating possesses the ability to enhance neovascularization in the vicinity of silicone implants. **Methods:** Plasma-treated collagen-I–coated silicone samples were placed into the dorsal skinfold chambers of female balb/c mice (*n* = 10). Conventional silicone served as control (*n* = 10). Intravital microscopy was performed within implant's surrounding tissue on days 1, 5, and 10. Functional vessel density, intervascular distance, vessel diameter, microvascular permeability, red blood cell velocity, and leukocyte-endothelium interaction were determined. **Results:** Enhanced angiogenesis in the tissue surrounding plasma-pretreated collagen-coated implants was noted. Significant increase of functional vessel density due to vascular new development was observed (*t* test, *P* < .05). Analyses of microvascular permeability and red blood cell velocity displayed stable perfusion of the vascular network neighboring the surface-modified implants. **Conclusion:** Intensified vascularity due to induced angiogenesis and neovascularization in the tissue surrounding plasma-collagen–coated samples were observed. These results indicate that plasma-mediated collagen coating might be a promising technology in order to improve the biocompatibility and versatility of silicone implants.

Silicone implants are often encapsulated by avascular fibrous tissue isolating them from the adjacent tissue. The fibrous capsule around the silicone implants, however, can lead to capsular contracture, one of the main complications after breast reconstruction and augmentation, which can cause painful indurations and implant extrusion making a surgical revision inevitable.^1^^-^3

The pathogenesis of fibrous encapsulation of implanted biomaterials is still uncertain. Several factors, such as filler material, implant placement, surface texture, and bacterial infection, have been accused to cause this pathological condition.^4^^-^6 Chemical, physical, and morphological characteristics of biomaterial surfaces are considered to play essential role in modulating cellular response at the tissue-/material interface.^7^,^8^ In view of this fact, great effort has been made to modify the interface of silicone implants by altering the surface texture to reduce the incidence of early capsular formation and contracture. However, previous studies indicate that alteration of the surface texture alone did not lead to a significant improvement of implants' biocompatibility.^9^^-^11

In contrast, bioactive surface coatings have emerged as a promising approach to improve the biocompatibility of such implants. Especially, collagen coating has been likened to an enhanced cell affinity to biomaterials. Therefore, this extracellular matrix protein has often been used in the field of biomedical and tissue engineering. Collagen, due to its cell-binding domain containing the amino acid's sequence arginine-glycine-asparagine, interacts with the cellular membrane via integrin receptors, and thereby influences cell growth, migration, differentiation, and adhesion.^12^,^13^ Coating of biomaterial surfaces with extracellular matrix components such as collagen has been noted to increase the vascularization of artificial scaffolds as well as the surrounding tissue of metallic implants.^14^,^15^

The lack of sufficient blood supply of the peri-implant tissue was recognized to limit the functionality of implants, especially, of those implants designed to release or detect molecules, such as drug delivery or sensing devices.^16^,^17^

Hence, an improved tissue-implant interaction leading to an enhanced formation of new blood vessels around the implant and decreasing the risk of capsule formation and subsequent capsular contraction is desirable. Biomimetic coatings that can be applied to implant surface to promote vascularization may thereby reduce the foreign body reaction and disrupt fibrous encapsulation.

However, an effective coating method of silicon surfaces that stimulates angiogenesis of the peri-implant tissue is currently not available.

Silicone used in plastic and reconstructive surgery is heat-sensitive and hydrophobic. Because of these characteristics, the coating of silicone, for example, with collagen remains difficult. Cold low-pressure gas plasma may possibly circumvent this obstacle. Cold plasma is a partially ionized low-pressure gas comprising ions, electrons, and ultraviolet photons, as well as reactive neutral species with sufficient energy to break covalent bonds on the material surface.^18^ Plasma treatment is a dry, cold (< 40°C), nontoxic and fast process, which makes it especially suitable for the treatment of vulnarable materials. Gas plasma does not cause structural damage of the implant but only interferes with the superficial layer of the material. The activation of the material surface via plasma treatment enhances the protein adhesion on the material; therefore, low-pressure plasma has the potential to provide bioactive protein coatings on implant surfaces.^19^,^20^

The aim of this study was to evaluate the biological response to plasma-mediated collagen-I–coated silicone implant material in vivo with special interest regarding the angiogenesis and neovascularization of the peri-implant tissue.

## MATERIALS AND METHODS

### Implant material

Standard texturized silicone implants for breast augmentation (Polytech Silimed, Deissenhofen, Germany) were used in both groups. The discoid implants had standardized dimensions (diameter, 2 mm; thickness, 300 µm).

The groups were as follows:

Group I: plasma pretreated and collagen-I–coated silcone samples (*n* = 10).

Group II: regular silicone implant material (non–plasma treated and non–collagen coated) served as control (*n* = 10).

### Plasma pretreatment

A double inductively coupled plasma reactor (Institute for Plasma Technology, Ruhr University Bochum, Germany) was used for coating experiments. The plasma was ignited and heated by an RF source at 13.65 MHz with a forward power of 1000 W. A gas mixture of argon (100 sccm) and oxygen (5 sccm) with a pressure of 10 Pa was used in this study. Each probe was treated for a duration of 5 minutes.

### Collagen-coating procedure

Collagen type I from rat tail 4 mg/mL in 20 mM acetic acid (BD Biosciences, Bedford, Mass) was diluted 1:8 with phosphate buffered saline to a final concentration of 0.5 mg/mL. After plasma treatment, 10 implants were incubated with 500 µL/cm^2^ for 24 hours at 4°C under sterile conditions. After 24 hours, the supernatant was discarded, and the probes were incubated for another 48 hours at 37°C. After drying, the implants were rinsed with phosphate-buffered saline and destilled water several times to wash off the nonadherent protein.

### Animals

The experiments were conducted in accordance with the European guidelines for the care and use of laboratory animals and German law for the protection of animals. The experiment comprised 20 female balb/c mice (Charles River, Sulzfeld, Germany) weighing from 18 to 22 g. The animals were caged individually. Tap water and standard laboratory food for mice (Sniff, Soest, Germany) were provided.

### Dorsal skinfold chamber

Using an operation stereomicroscope, 2 titanium chamber frames were implanted to sandwich the stretched skinfold of animal's back. One layer of skin was completely excised in a circular fashion, and the remaining striated skin muscle layer of the opposite side was covered with a coverslip.^21^

Forty-eight hours after the surgery, the implants were placed in direct contact to the perfused striated skin muscle in the center of the chamber. The chamber was then closed using a new sterile coverslip.

### Intravital fluorescence microscopy

Microscopic observation was performed on days 1, 5, and 10 postimplantation. Standard intravital microscopy was used for microcirculatory observations after intravenous injection of 50 µL of 1% FITC (fluorescein-isothiocyanate)-labeled Dextran (MW 150.000) and 50 µL of 0.5% Rhodamin G6 (both: Sigma Chemicals, Deisenhofen, Germany) via tail vein. An intravital microscope with a 40-fold water immersion objective (Zeiss, Axiotech Vario 100 HD, Achroplan 20 × 0.5 W, Zeiss, Oberkochen, Germany) was used to examine the microvasculature.

Three different regions of interest were defined within the tissue immediately surrounding the implants and recorded and outlined using the Axiovision 3.1 system (Carl-Zeiss-Vision GmbH, Oberkochen, Germany) for exact relocation. At the end of the protocol, the implants were harvested for histological examination.

### Data acquisition

Analysis of the intravital video imaging was performed by using the computer program CapImage (Dr Zeintl, Heidelberg, Germany). Functional vessel density (FVD in cm/cm^2^), intervascular distance (µm), vessel diameter (VD in µm), microvascular permeability (Ie/Ii), red blood cell velocity (mm/s), and leukocyte-endothelium interaction expressed by the number of adherent leukocytes (n/mm^2^) were measured for the assessment of microcirculatory changes.^22^ Statistical analysis was performed using *t* test (*P* < .05 was regarded as statistically significant) using SigmaStat (SPSS Inc, Version 2.03, Chicago, Ill).

## RESULTS

**Figure 1:**
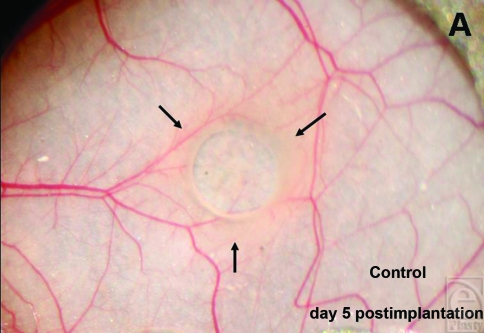

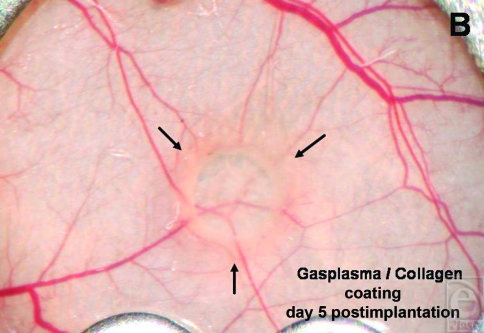

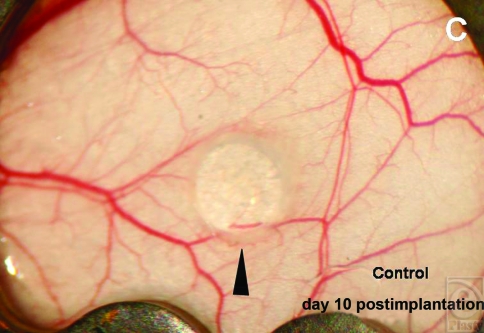

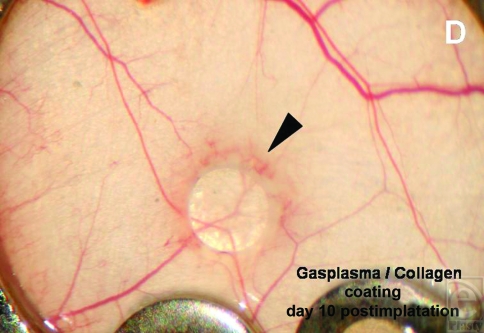
The images show an edema wall (arrows) that developed around untreated (*a*) and plasma-pretreated collagen-coated (*b*) silicone implants on day 5 postimplantation (Implant diameter = 2 mm). On day 10 postimplantation, the muscle tissue surrounding the coated implants (*d*) show a prominent hyperemic hem (triangle). The hyperemia of the adjacent tissue of untreated implant is less intensive (*c*).

Macroscopically a restricted zone of edema formation around the implants could be seen from day 5 (Figs 1a and 1b). On day 10, the tissue surrounding the plasma-collagen–coated silicone implants showed a noticeable hyperemic hem, which was prominent in treated group compared with controls (Figs 1c and 1d).

**Figure 2:**
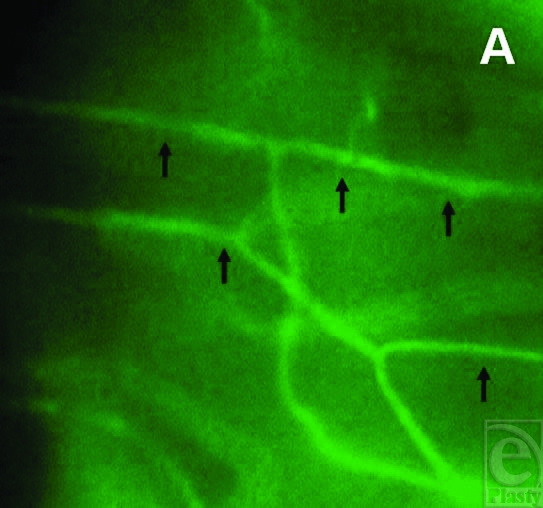

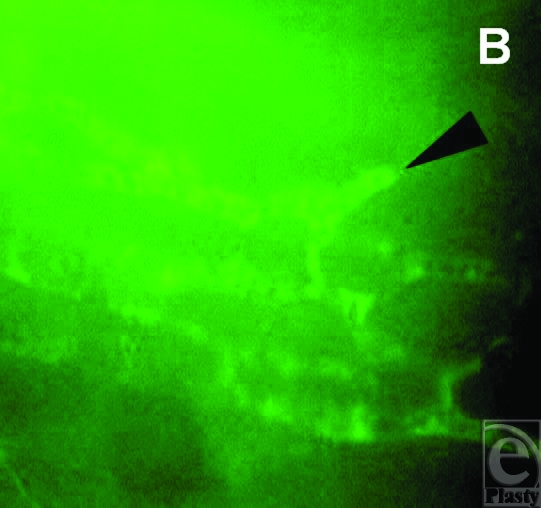

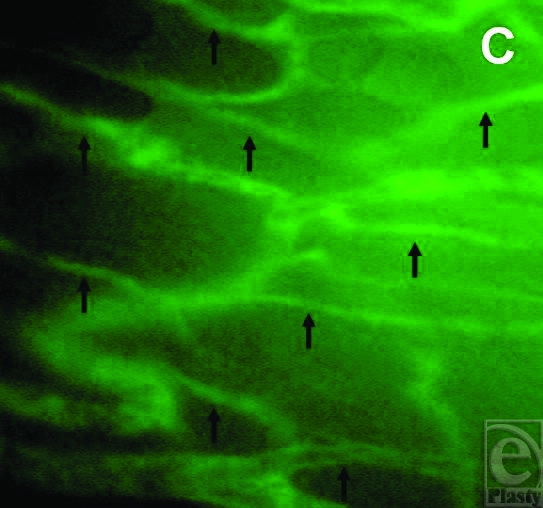
The intravital fluorescence images of the border zone of plasma-collagen–coated silicone implants show normal arranged capillaries of the striated skin muscle on day 1 postimplantation (*a*). Dilated and perfused capillaries bearing vessel sprouts (triangle) surrounded by a pronounced paravascular edema formation due to extravasation of plasma marker FITC (fluorescein-isothiocyanate)-Dextran were detected on day 5 (*b*). Increased vascular density due to vessel new development and remodeling of the vascular network were noted on day 10 (*c*) (magnification: 350-fold).

**Figure 3:**
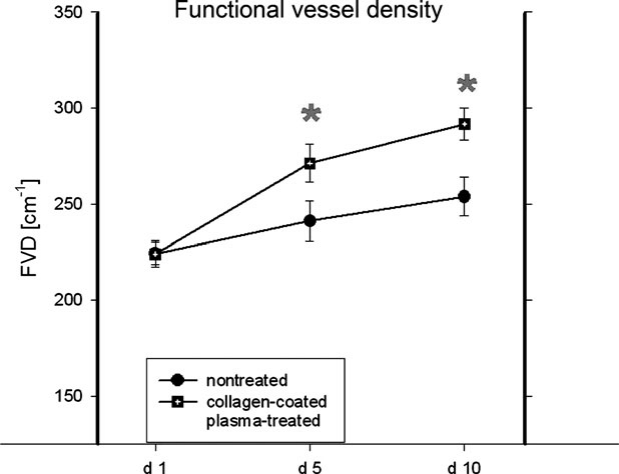

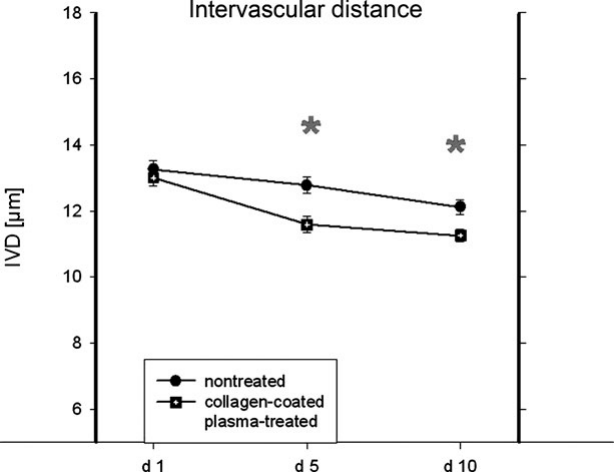

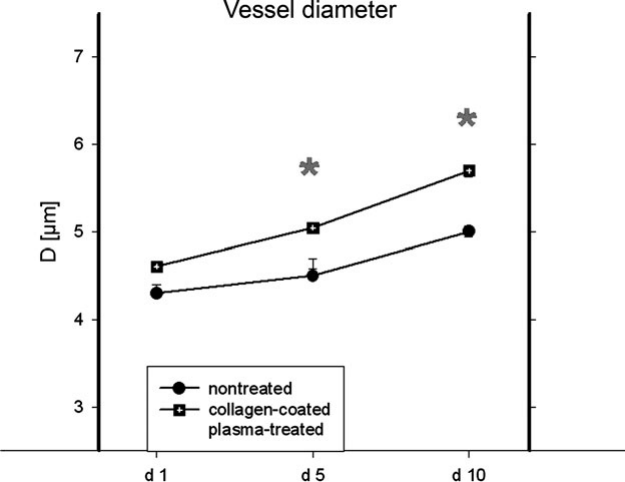

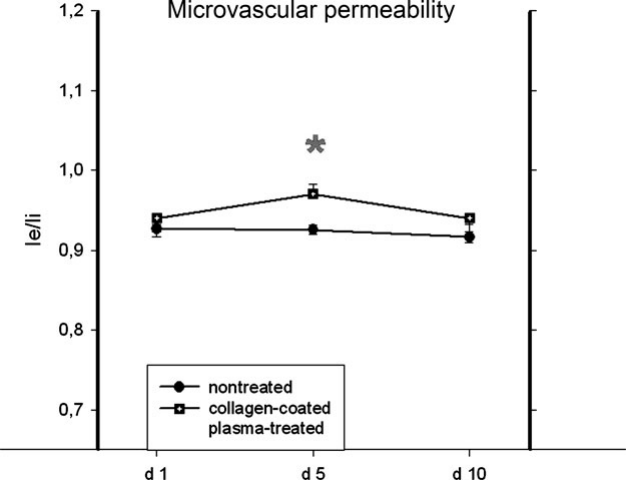

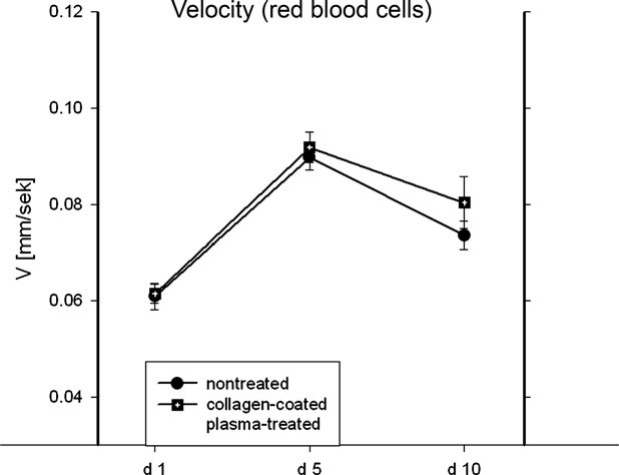

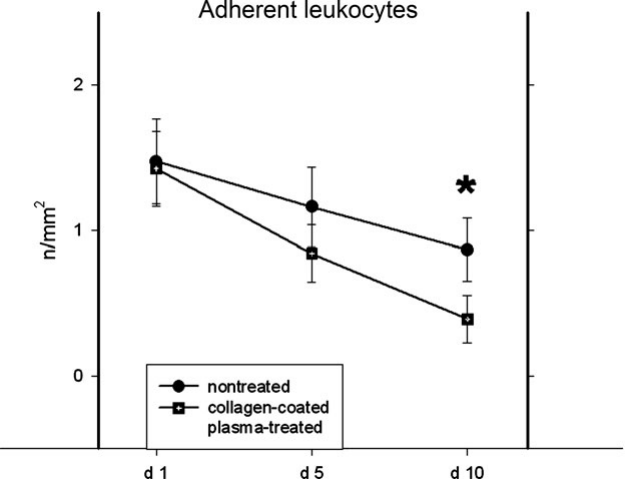
The graphs display the quantification of standard microcirculatory parameters. (*a*) Increasing functional (perfused) vessel density (FVD) was noted within the border zones of untreated and coated implants throughout the experiment. The differences among the groups reached statistical significance on days 5 and 10. (*b*) The assessment of vessel diameter (VD) demonstrated significantly increased values for the treatment group on days 5 and 10. (*c*) The examination of the intervascular distance demonstrated a progressive and significant reduction from day 1 to day 10 in both groups. (*d*) The analyses of red blood cell velocity revealed a stable perfusion of the blood-vessel network in implant adjacent tissue. (*e*) The microvascular permeability was found to be significantly increased on day 5 in treated implant group as compared with controls. (*f*) The examination of the leukocyte-endothelium interaction showed that the quantity of leukocytes adhering to vessel walls decreased from day 1 to day 10. Significant differences between the groups were found on day 10 (*t* test, *P* < .05).

The intravital microscopy of the peri-implant tissue reinforced this observation, where an increase in vessel diameter of perfused microvessels, new vessel development, and raised vascular density in the treated implant group was noted on days 5 and 10, respectively (Figs 2a–2c). On day 5, the FVD in the border zone of collagen-coated plasma-treated implants was 271 ± 9.8 cm/cm^2^ and around 291 ± 8.5 cm/cm^2^ on day 10 on average (mean ± SEM). In comparison, the FVD in the border zone of the untreated implants averaged around 241 ± 10.4 cm/cm^2^ on day 5 and 253 ± 10 cm/cm^2^ on day 10 (Fig 3a). The differences among the groups reached statistical significance. In addition, the vessel diameters displayed significantly increased values for the treatment group on days 5 and 10 compared with controls (Fig 3b).

The examination of the intervascular distance demonstrated a progressive and significant reduction from day 1 to day 10 in both groups (Fig 3c). The analyses of red blood cell velocity and microvascular permeability revealed resolute perfusion and stability of newly developed blood-vessel network around the collagen-coated plasma-treated implants (Figs 3d and 3e). The microvascular permeability was found to be significantly increased on day 5 in treated implant group as compared with controls.

**Figure 4:**
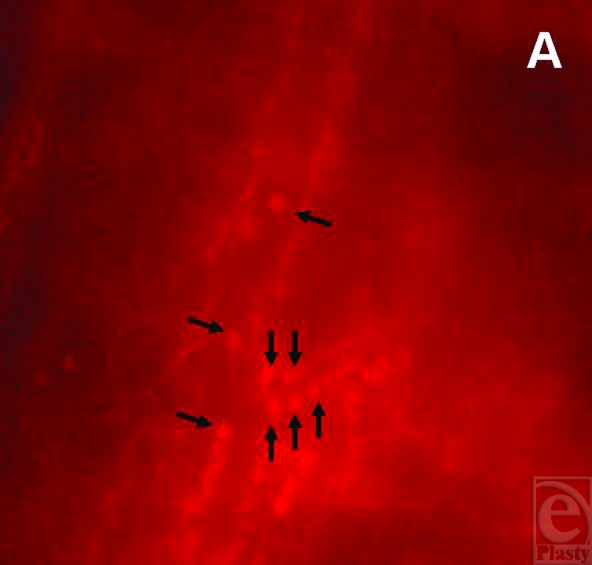

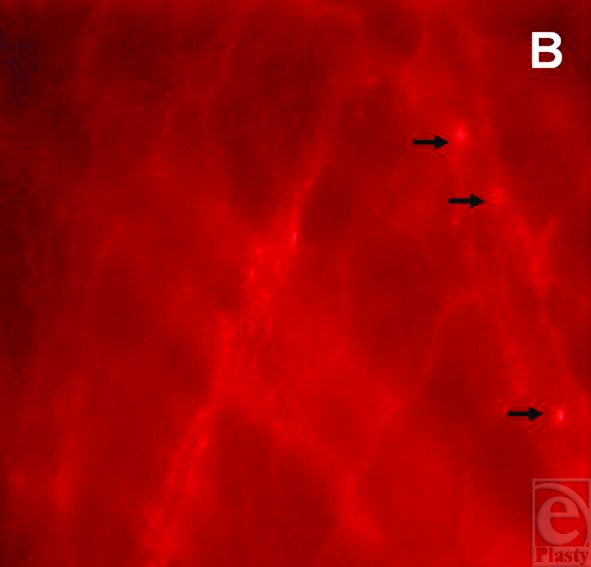
The intravital fluorescence images display the leukocyte-endothelium interaction on day 1 (*a*) and day 10 (*b*) within border zone of plasma-collagen–coated silicone implants. A quantitative reduction of leukocytes adhering to vessel walls of a postcapillary venule (arrows) was observed throughout the experiment (magnification: 350-fold).

The examination of the leukocyte-endothelium interaction showed that the quantity of leukocytes adhering to vessel walls decreased over the observation period (Figs 4a and 4b). Significant differences between the groups were found on day 10 (Fig 3f).

**Figure 5:**
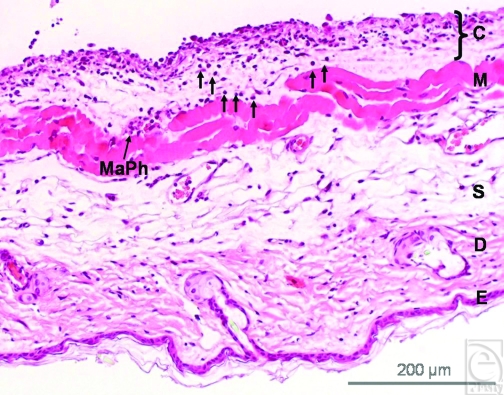

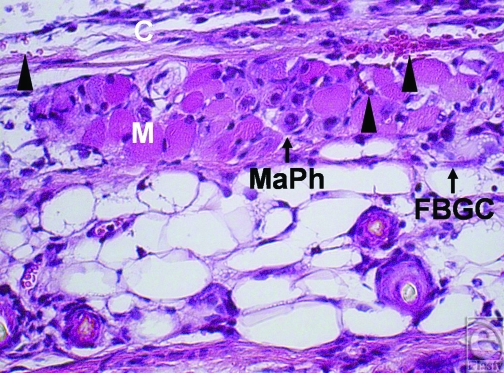

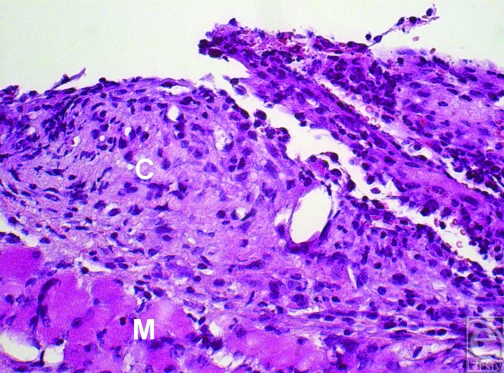
(*a*) The image shows the cross-section of a skin tissue sample (hematoxylin and eosin staining) that was exposed to plasma-treated collagen-coated silicone implant. (E = epidermis, D = dermis, S = subcutis, M = striated skin muscle, C = capsule). Leukocytes (arrows) penetrate the skin muscle layer (M) directed to the capsular tissue (C). Macrophages (MaPh) are evident at the capsule-muscle interface (magnification: 100-fold). (*b*) An extensive fibrous deposition within the implant capsule and enhanced infiltration of the capsular tissue by leukocytes were found in the untreated group (magnification: 200-fold). (*c*) The muscle and the fibrous capsular layer of treated implants show a high vascular infiltration (blood vessels are marked by triangles). Macrophages (MaPh) and a foreign-body giant-cell (FBGC) are detected within the muscle layer. The inflammatory cells are directed towards implant's capsule (magnification: 200-fold).

The histological examination of the samples showed few inflammatory cells penetrating into the surrounding skin muscle tissue in the treated implant group (Fig 5a). On the contrary, a noticeable accumulation of inflammatory cells, mainly leukocytes and macrophages, as well as few foreign body giant cells, was detected at the implant-tissue interface in the untreated implant group. In addition, the fibrous deposition was found more pronounced in untreated group (Fig 5b) whereas the muscle and the fibrous layer in treated implants displayed a higher vascular content (Fig 5c).

## DISCUSSION

An improved surface hydrophilicity due to gas plasma treatment was demonstrated for various synthetic implant materials, for example, PET, PTFE, and poly(lactide-co-glycolide), whereby enhanced attachment and proliferation of human skin–derived fibroblasts, the growth of human endothelial cells, and platelets were found. An enhanced cell adhesion on poly(D,L-lactide) was also shown for the combination of gas plasma surface pretreatment with collagen coating.^23^^-^26

One of the first attempts to increase the biocompatibility of silicone implant materials by surface coating with collagen was made by Ksander et al. This collagen coating led to a significantly reduced capsule formation. However, the collagen was cross-linked with formaldehyde and glutaraldehyde. Due to cytotoxic and carcinogenic properties of these chemical additives, this surface treatment turned out to be unsuitable for in vivo use.^27^,^28^

Increased collagen adsorption and improved collagen adhesion to plasma-treated implant surfaces was later shown by Gölander et al,^29^ who proved that the extent of surface-free energy is a decisive factor in terms of the protein-binding capacity of an implant surface. Baier et al^30^ described that a high level of surface-free energy improves the adsorption of hydrophilic proteins such as collagen. The plasma-induced enhanced hydrophilicity of silicone led to optimization of collagen binding on implants surface.

Previous in vitro cell experiments revealed that cell adhesion and cell viability of murine fibroblasts is significantly enhanced on plasma-activated and collagen-coated silicone implants.^26^ Besides, it was observed that the surface modification by gas plasma influenced the progress of new vessel formation and promoted vascular infiltration in polymer scaffolds.^21^

In the current study, a significant increase of neovascularization within tissue adjacent to the silicone implants when pretreated with cold low-pressure plasma and coated with collagen was detected. The peri-implant tissue of treated silicone material showed an intensified microvascular growth. During the observation period, an increased perfusion of the new-developed vascular network around collagen-coated plasma-treated implants was noted as quantified by standard microcirculatory parameters as functional vessel density, vessel diameter, and flow velocity. The enhanced vascular density of the peri-implant tissue in plasma-treated collagen-coated group as assessed by intravital microscopy was associated with a higher vascular content within the muscle layer at the implant-/tissue interface in histology.

In contrast to the treated silicone implants, the implant-/tissue interface in the control group revealed histologically a pronounced infiltration of inflammatory cells especially as leukocytes. Although the presence of macrophages at the implant-/tissue interface was noted in both groups, the macrophages appeared to accumulate to a higher degree in the untreated implant group. On the contrary, the intravital quantification of leukocytes adherent to vessel walls of postcapillary venules, however, did not reflect the histological findings. The number of sticking leukocytes was not elevated but rather decreased over the incubation period of 10 days in both groups. An intensive accumulation of adherent leukocytes, which could have explained the histological finding of a high rate of penetration into the muscle and capsule tissue by inflammatory cells were not found during intravital microscopy.

The biomaterial surface may activate inflammatory cells and modulate their cytokine and chemokine release. Macrophage-derived cytokines are known to activate leukocytes, which increase the rate of monocyte adhesion and fusion. Especially macrophages guide the foreign body response thus leading to a fibrous encapsulation of the implant.^31^^-^33 Jones et al^34^ demonstrated that material surface has impact on macrophage-derived secretion of matrix metalloproteinases (MMPs) and their tissue inhibitors (TIMPs). It was found that macrophages while activated by material surface produce a greater quantity of angiogenesis-associated factors such as MMP-9, TIMP-1, and TIMP-2. Besides these findings, there are indications that the elevated systemic concentration of tissue inhibitors of metalloproteinases is associated with the pathogenesis of fibrosis after breast augmentation with silicone implants. Ulrich et al^35^ found that the balance between MMPs and TIMPs is disturbed in patients with capsule contracture thus causing alteration in synthesis and deposition of collagen surrounding implants.^36^

In the current study, the extravasation of the plasma marker FITC-Dextran, however, showed that the edema formation in perivascular space was only a transient phenomenon in collagen-coated plasma-treated group. The increased microvascular permeability found on day 5 postimplantation as compared with control was not associated with a prolonged loss of vascular integrity.

The reduction of vascular integrity leading to increased permeability is a common finding during angiogenesis but may also be observed as perivascular edema due to plasma exudation during inflammation. Such inflammatory effects may be exerted by activation of G-protein–coupled receptors such as cysteinyl leukotrienes receptors, which mediate the constriction of vascular smooth muscle cells.^37^ By the way, D'Andrea et al^38^ detected an increase in cysteinyl leukotrienes receptors gene expression on macrophages in periprosthetic capsular tissue of patients affected by capsular contracture.^39^

Summarizing, the model used for the current study offered a direct in vivo visualization of microcirculation and assessment of microvascular and inflammatory situation within peri-implant tissue.

The results presented indicate that plasma-mediated collagen coating may be a promising strategy to increase vascularity around implants thus enhancing the biocompatibility and the versatility of silicone implant material.

Certainly, the findings made over a period of 10 days cannot be blindly extrapolated to a long-term in vivo situation. The intravital results reflect just snapshots of a short-term observation. Nevertheless, the analysis at the implant-tissue interface allowed for determination of the initial course of the host tissue response in terms of early inflammatory reaction and angiogenesis.

Thus, the induction of angiogenesis and an increased neovascularization in adjacent tissue of collagen-coated plasma-treated silicone implant materials may indicate a continuing tissue response. Whether the plasma-mediated collagen coating will permit prolonged inhibition of an avascular capsule formation is unknown. The fact that the neovascularization of the tissue in close proximity to treated implants increased and the microcirculation remained stable throughout the experiment raises the promise of a reduced risk for capsular fibrosis. Further long-term studies are needed to confirm this assumption.

## Acknowledgments

The authors sincerely thank Institute for Plasma Technology at the Ruhr University Bochum for expert technical assitance. This work is part of the doctoral thesis of Lena Henrich.
